# Glutamine involvement in nitrogen regulation of cellulase production in fungi

**DOI:** 10.1186/s13068-021-02046-1

**Published:** 2021-10-13

**Authors:** Ai-Ping Pang, Funing Zhang, Xin Hu, Yongsheng Luo, Haiyan Wang, Samran Durrani, Fu-Gen Wu, Bing-Zhi Li, Zhihua Zhou, Zuhong Lu, Fengming Lin

**Affiliations:** 1 State Key Laboratory of Bioelectronics, School of Biological Science and Medical Engineering, Southeast University, Nanjing, China; 2 Key Laboratory of Systems Bioengineering (Ministry of Education), School of Chemical Engineering and Technology, Tianjin University, Tianjin, China; 3 Key Laboratory of Synthetic Biology, Institute of Plant Physiology and Ecology, Shanghai Institutes for Biological Sciences, Chinese Academy of Sciences, Shanghai, China

**Keywords:** Glutamine, Cellulase, Nitrogen metabolism, The TOR pathway, ooc1, trFKBP12

## Abstract

**Background:**

Cellulase synthesized by fungi can environment-friendly and sustainably degrades cellulose to fermentable sugars for producing cellulosic biofuels, biobased medicine and fine chemicals. Great efforts have been made to study the regulation mechanism of cellulase biosynthesis in fungi with the focus on the carbon sources, while little attention has been paid to the impact and regulation mechanism of nitrogen sources on cellulase production.

**Results:**

Glutamine displayed the strongest inhibition effect on cellulase biosynthesis in *Trichoderma reesei*, followed by yeast extract, urea, tryptone, ammonium sulfate and l-glutamate. Cellulase production, cell growth and sporulation in *T. reesei* RUT-C30 grown on cellulose were all inhibited with the addition of glutamine (a preferred nitrogen source) with no change for mycelium morphology. This inhibition effect was attributed to both l-glutamine itself and the nitrogen excess induced by its presence. In agreement with the reduced cellulase production, the mRNA levels of 44 genes related to the cellulase production were decreased severely in the presence of glutamine. The transcriptional levels of genes involved in other nitrogen transport, ribosomal biogenesis and glutamine biosynthesis were decreased notably by glutamine, while the expression of genes relevant to glutamate biosynthesis, amino acid catabolism, and glutamine catabolism were increased noticeably. Moreover, the transcriptional level of cellulose signaling related proteins ooc1 and ooc2, and the cellular receptor of rapamycin trFKBP12 was increased remarkably, whose deletion exacerbated the cellulase depression influence of glutamine.

**Conclusion:**

Glutamine may well be the metabolite effector in nitrogen repression of cellulase synthesis, like the role of glucose plays in carbon catabolite repression. Glutamine under excess nitrogen condition repressed cellulase biosynthesis significantly as well as cell growth and sporulation in *T. reesei* RUT-C30. More importantly, the presence of glutamine notably impacted the transport and metabolism of nitrogen. Genes *ooc1*, *ooc2*, and *trFKBP12* are associated with the cellulase repression impact of glutamine. These findings advance our understanding of nitrogen regulation of cellulase production in filamentous fungi, which would aid in the rational design of strains and fermentation strategies for cellulase production in industry.

**Supplementary Information:**

The online version contains supplementary material available at 10.1186/s13068-021-02046-1.

## Background

In nature, organisms sense nutrient availability in the surrounding environment and adjust their metabolism for optimal growth, development, and reproduction [1]. Carbon and nitrogen are two of major elements required for life. In fungi, nitrogen metabolism is controlled by a complex genetic regulatory circuit which ensures the preferential use of primary nitrogen sources (ammonium and glutamine) and also confers the ability to use many different secondary nitrogen sources (i.e., nitrate) when appropriate [2–6], which is similar to carbon catabolite repression (CCR). Most genes encoding nitrogen catabolic enzymes are subject to nitrogen catabolite repression (NCR), mediated by positive-acting transcription factors of the GATA family of proteins. The standard model of NCR is that AreA mediates de-repression of genes for utilization of secondary nitrogen sources in the absence of ammonium or glutamine. The quality and quantity of nitrogen impact the biosynthesis of many known secondary metabolites in fungi [7, 8], such as cellulase in *Penicillium occitanis* [9] or *Penicillium funiculosum* [10], gibberellin in *Gibberella fujikuroi* [11], deoxynivalenol in *Fusarium graminearum* [12], and aflatoxin in *Aspergillus flavus* [13]. Study has been making progress on the effect and regulation mechanism of nitrogen sources on cellulase production.

Nitrogen metabolism plays an important role in cellulase production in filamentous fungi like *T. reesei*, *Aspergillus nidulans*, and *P. funiculosum*. In *T. reesei*, deletion of the global nitrogen regulator Are1 decreased cellulase production when using (NH_4_)_2_SO_4_ as the nitrogen source, demonstrating a role of Are1 in the regulation of cellulase production [14]. In *A. nidulans*, cellulase production is impacted by both carbon and nitrogen source, and is under the regulation of the carbon regulators CreA, CreB, and CreC, as well as the nitrogen regulator AreA, either directly or indirectly [15]. Meanwhile, efforts have been put on optimizing nitrogen sources for cellulase production. A sequential experimental design methodology has been explored for nitrogen source optimization for the enhancement of cellulase production in *P. funiculosum* by evaluating various nitrogen sources including ammonium sulfate, urea, yeast extract, and peptone [10]. Suitable nitrogen sources for cellulase production have been described for *T. reesei* [16, 17]. *T. reesei* can consume a variety of nitrogen-containing compounds with little change in cellulase production, while nitrogen limitation inhibited cell growth and cellulase biosynthesis [17]. However, compared to the extensive research on the carbon regulation on cellulase production [18–25], less attention has been paid to nitrogen regulation of cellulase production with the underlying mechanism unknown.

To investigate the nitrogen regulation of cellulase production in fungi, we evaluated the influence of various nitrogen sources on cellulase production under excess complex nitrogen condition, finding that glutamine exhibited the strongest inhibition effect on cellulase biosynthesis. Then the effect of glutamine on *T. reesei* RUT-C30 grown under cellulase-producing condition was investigated in terms of cellulase synthesis, cell growth, sporulation ability, and morphology. The molecular mechanism behind glutamine-repressed cellulase production was explored by comparative transcriptional profiling and the knockout of genes *ooc1*, *ooc2*, and *trFKBP12*.

## Results

### Glutamine exhibited stronger inhibition effect on cellulase biosynthesis than other nitrogen sources under excess complex nitrogen sources

**Fig. 1:**
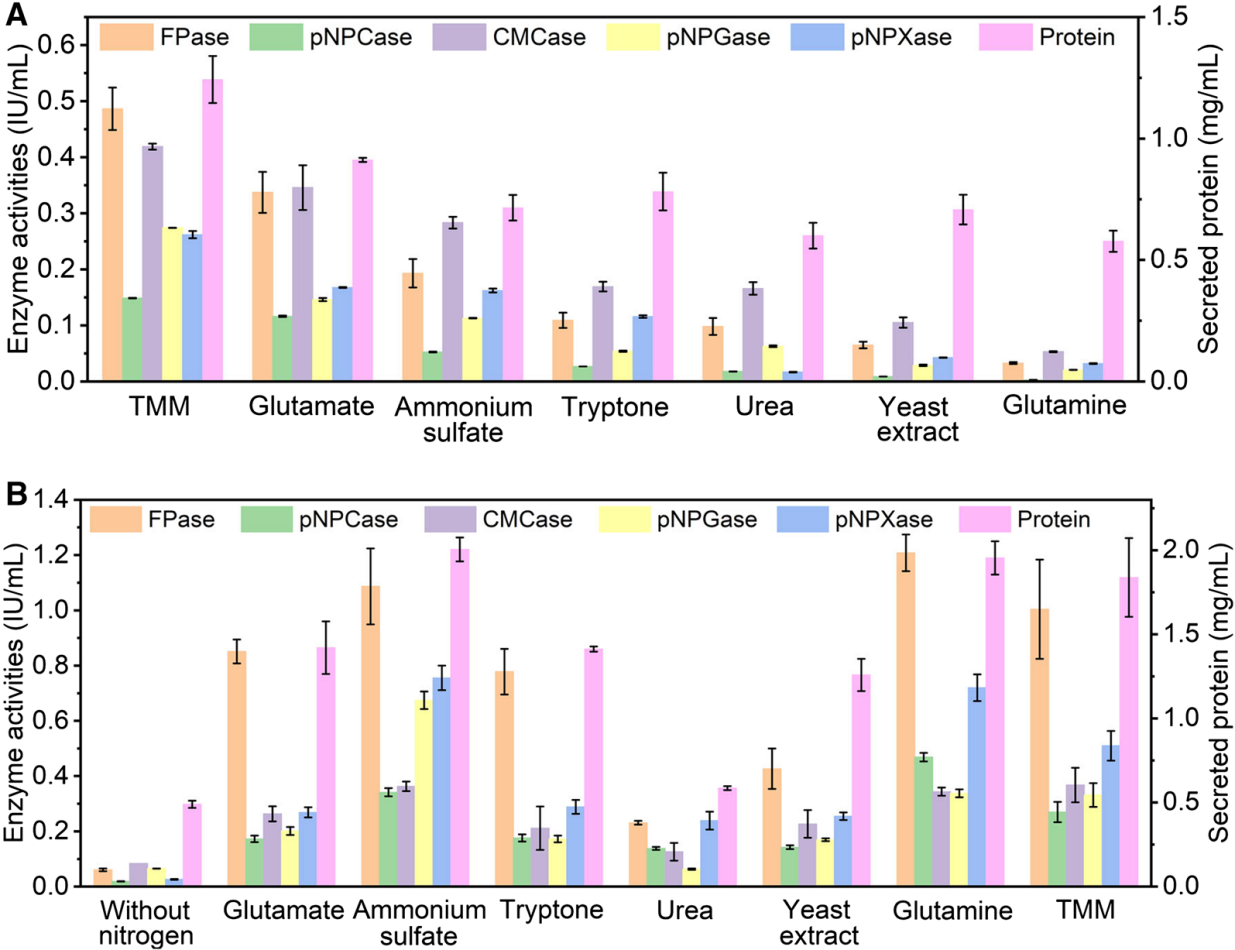
Effect of different nitrogen sources on (hemi)cellulase activities and protein secretion of *T. reesei* RUT-C30 at 72 h. **A** Enzyme activities of *T. reesei* RUT-C30 cultured in TMM with the individual addition of different nitrogen sources of 2.92 g/L. **B** Enzyme activities of *T. reesei* RUT-C30 cultured with different nitrogen sources as a sole nitrogen source at 4 g/L. FPase: the filter paper activity; pNPCase: the CBH activity; CMCase: the CMC activity; pNPGase: the β-glucosidase activity; pNPXase: the β-xylosidase activity; Secreted protein: secreted protein concentration. Data are represented as the mean of three independent experiments and error bars express the standard deviations

First, we explored the impact of the addition of equal amount of varied nitrogen sources into *Trichoderma* minimal media (TMM) on cellulase production, including glutamine, glutamate, ammonium sulfate, yeast extract, tryptone, and urea (Fig. 1A). The addition of different nitrogen sources at 2.92 g/L inhibited enzyme activities and protein secretion to varying degrees, of which glutamine had the most severe inhibition effect, followed by yeast extract, urea, tryptone, ammonium sulfate and glutamate. In the presence of glutamine, the FPase, CMCase, pNPCase, pNPGase and pNPXase activities and secreted protein at 72 h were decreased by 93.27%, 98.22%, 87.33%, 92.48%, 87.81%, and 53.54%, respectively, as compared to those of RUT-C30 cultured in only TMM. Clearly, the inhibition effect on cellulase biosynthesis was found for all the tested types of nitrogen sources, indicating that the addition of more nitrogen into TMM caused nitrogen excess that compromised the cellulase production (given that TMM already contains complex nitrogen sources including 4 g/L ammonium sulfate, 1 g/L urea, 0.75 g/L tryptone, and 0.25 g/L yeast extract). On the other hand, the huge difference on cellulase production repression between glutamate and glutamine suggested that the extent of cellulase inhibition is also dependent on the types of nitrogen sources. Meanwhile, this result demonstrated that cellulase production was much more sensitive to glutamine than other nitrogen sources under excess nitrogen conditions.

When using each tested nitrogen source of 4 g/L as the sole nitrogen source to replace all the nitrogen sources in TMM, excellent cellulase activities were observed for most of the nitrogen sources, except urea (Fig. 1B). The highest (hemi)cellulase activities were found on glutamine, ammonium sulfate, or glutamate, followed by tryptone, yeast extract, and urea (Fig. 1B).

### High dose glutamine exerted a repression on (hemi)cellulase production in *T. reesei*

**Fig. 2:**
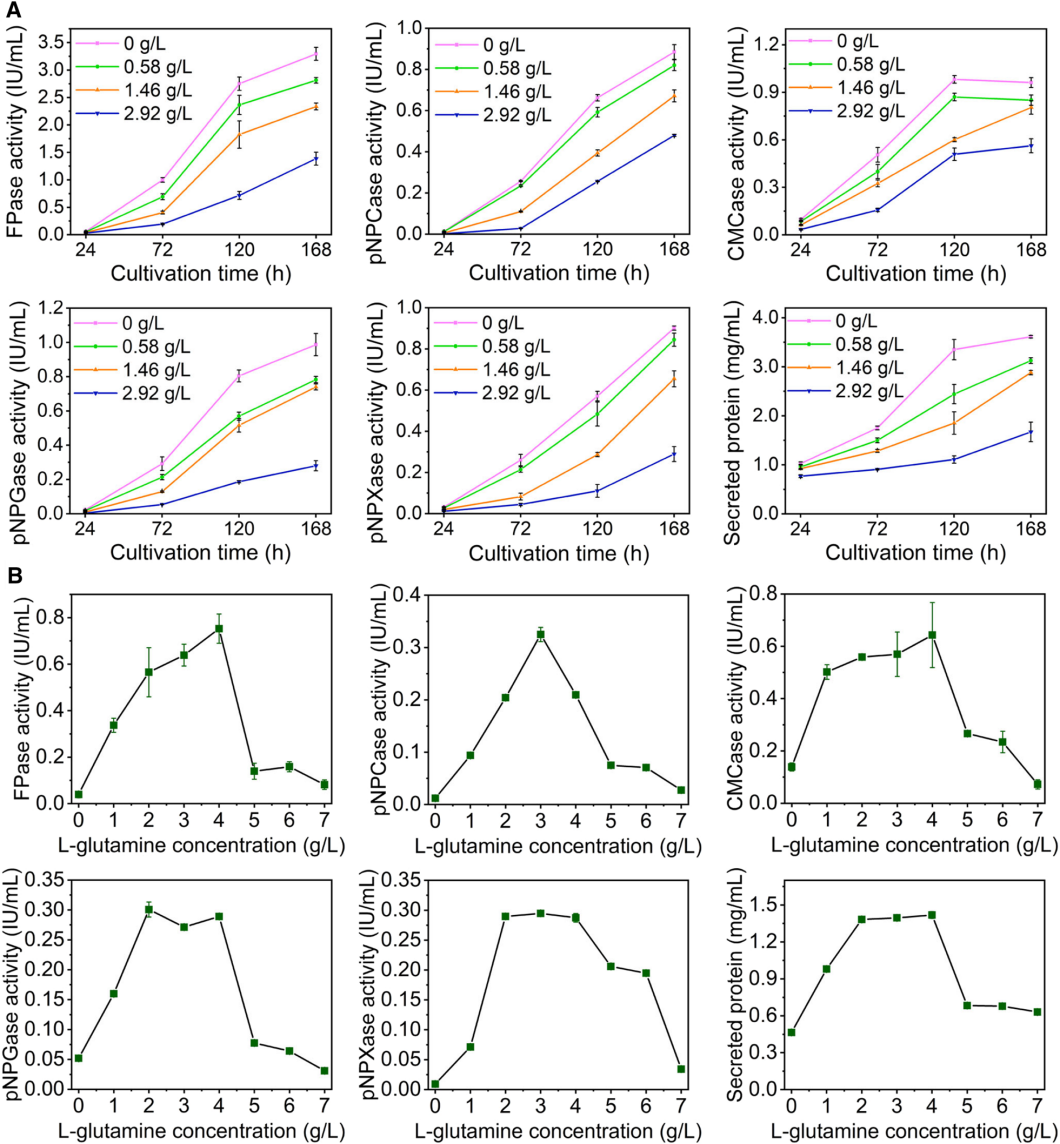
Effect of glutamine on (hemi)cellulase activities and protein secretion of *T. reesei* RUT-C30. **A** Enzyme activities of *T. reesei* RUT-C30 cultured in TMM with the supply of glutamine at varied concentrations. **B** Enzyme activities of *T. reesei* RUT-C30 cultured with different concentrations of glutamine as a sole nitrogen source. FPase: the filter paper activity; pNPCase: the CBH activity; CMCase: the CMC activity; pNPGase: the β-glucosidase activity; pNPXase: the β-xylosidase activity; Secreted protein: secreted protein concentration. Data are represented as the mean of three independent experiments and error bars express the standard

In detail, the effect of glutamine on (hemi)cellulase production of *T. reesei* RUT-C30 under cellulase-producing condition was further investigated by adding different physiological levels of glutamine (0, 0.58, 1.46, and 2.92 g/L) into TMM + 2% cellulose (Fig. 2A). As the concentration of glutamine was increased from 0.58 to 2.92 g/L, the increasing rate of (hemi)celluase activities and secreted protein concentration along the fermentation time were lowered continuously, showing that the (hemi)cellulase production was repressed by the treatment of glutamine. The addition of 2.92 g/L glutamine severely blocked cellulase production, leading to a decline by 63.0%, 50.8%, 33.7%, 71.6%, and 67.9% for FPase, pNPCase, CMCase, pNPGase and pNPXase activities, respectively, at 168 h. However, this inhibition effect was not complete, for cellulase activities and secreted protein concentration were still raised at a very low speed as the fermentation progressed even in the presence of 2.92 g/L glutamine. These findings indicate that the supplementation of glutamine into TMM slowed down the (hemi)cellulase production in *T. reesei* profoundly.

Moreover, glutamine with various concentrations were used as the sole nitrogen sources in TMM to produce cellulase in *T. reesei* (Fig. 2B). The (hemi)cellulase activities at 72 h first rose and then decreased as the concentration of glutamine increased (Fig. 2B). The highest enzyme activity occurred at 4 g/L glutamine for FPase and CMCase, at 3 g/L for pNPCase, and at 2–4 g/L for pNPGase and pNPXase. The effect of glutamine on the secreted protein concentration followed a similar trend to pNPGase and pNPXase activities. When the glutamine concentration was beyond 4 g/L that was much lower than the total amount of nitrogen sources in TMM (6 g/L), reduced cellulase activities were observed, implying that (hemi)cellulase production was more sensitive to glutamine excess than the nitrogen complex commonly used in TMM. Obviously, the impact of glutamine on cellulase production was concentration-dependent, serving as an inducer for cellulase production at low concentration but an inhibitor at high concentration. All these demonstrated that glutamine exerted a significant inhibition on cellulase biosynthesis under nitrogen excess, and the decreased cellulase production by the addition of glutamine in TMM (Fig. 1) was due to both surplus nitrogen source and glutamine inhibition.

### The phenotype effect of glutamine on *T. reesei*

**Fig. 3:**
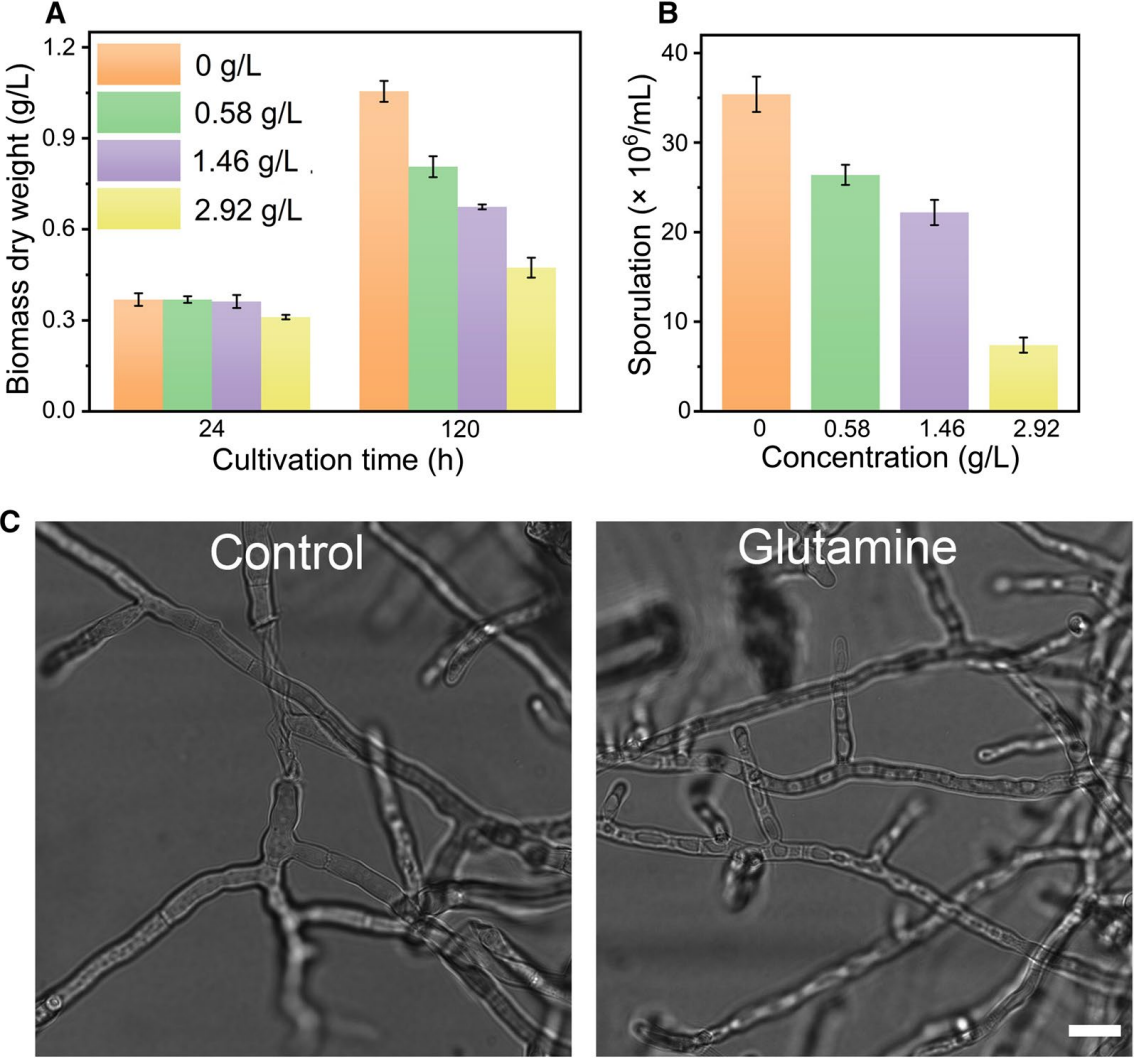
Effect of glutamine on **A** growth, **B** sporulation at 120 h and **C** morphology at 24 h of *T. reesei* RUT-C30 grown on TMM with the addition of glutamine at varied concentrations. The biomass dry weight per liter was calculated from the intracellular protein content based on 0.32 g of intracellular protein per g dry biomass. Error bars show the respective standard deviation of three biological replicates. Scale bar = 10 μm

Next, we investigated the effect of glutamine on growth, sporulation and morphology of *T. reesei* RUT-C30 grown on cellulose (Fig. 3). At 24 h, no significant difference of growth was observed until the concentration of glutamine was increased to 2.92 g/L compared to *T. reesei* without glutamine. At 120 h, the inhibition effect of glutamine became obvious with 23.5%, 36.1%, and 55.1% growth reduction in the presence of 0.58 g/L, 1.46 g/L, and 2.92 g/L glutamine, respectively (Fig. 3A). The sporulation ability of *T. reesei* was also retarded with the treatment of glutamine. The spore amount of *T. reesei* was gradually decreased when the concentration of glutamine was increased (Fig. 3B). In the presence of 2.92 g/L glutamine, the spore amount was only 20.9% of that of non-treated RUT-C30. Nevertheless, the morphology of RUT-C30 treated with 2.92 g/L glutamine was similar to that of non-treated RUT-C30 (Fig. 3C), indicating that glutamine does not affect the morphology of *T. reesei*. These results suggested that the addition of glutamine impaired growth and sporulation of *T. reesei* on cellulose, but not its morphology.

### The effect of glutamine on the transcriptome of *T. reesei*

Transcriptome sequencing analysis was carried out using *T. reesei* RUT-C30 cultured in TMM plus 2% cellulose with/without 2.92 g/L glutamine for 24 h to understand how glutamine influences *T. reesei* at the transcriptional level. The sequences of the total reads were mapped to the reference genome of *T. reesei* RUT-C30 (https://www.ncbi.nlm.nih.gov/genome/323%3fgenomeassembly_id%3d49799) with coverage of 93.98–94.51%. A total of 10,048 unique transcripts were detected (Additional file 1: Table S1). Genes were differentially expressed between the two strains when the average reads of the corresponding transcripts differed with |log_2_Ratio|≥ 1 and adjusted *p* values ≤ 0.05. In the presence of glutamine, 1192 differentially expressed genes (DEGs) were obtained, of which 699 were upregulated and 493 were downregulated (Additional file 2: Table S2).

**Fig. 4:**
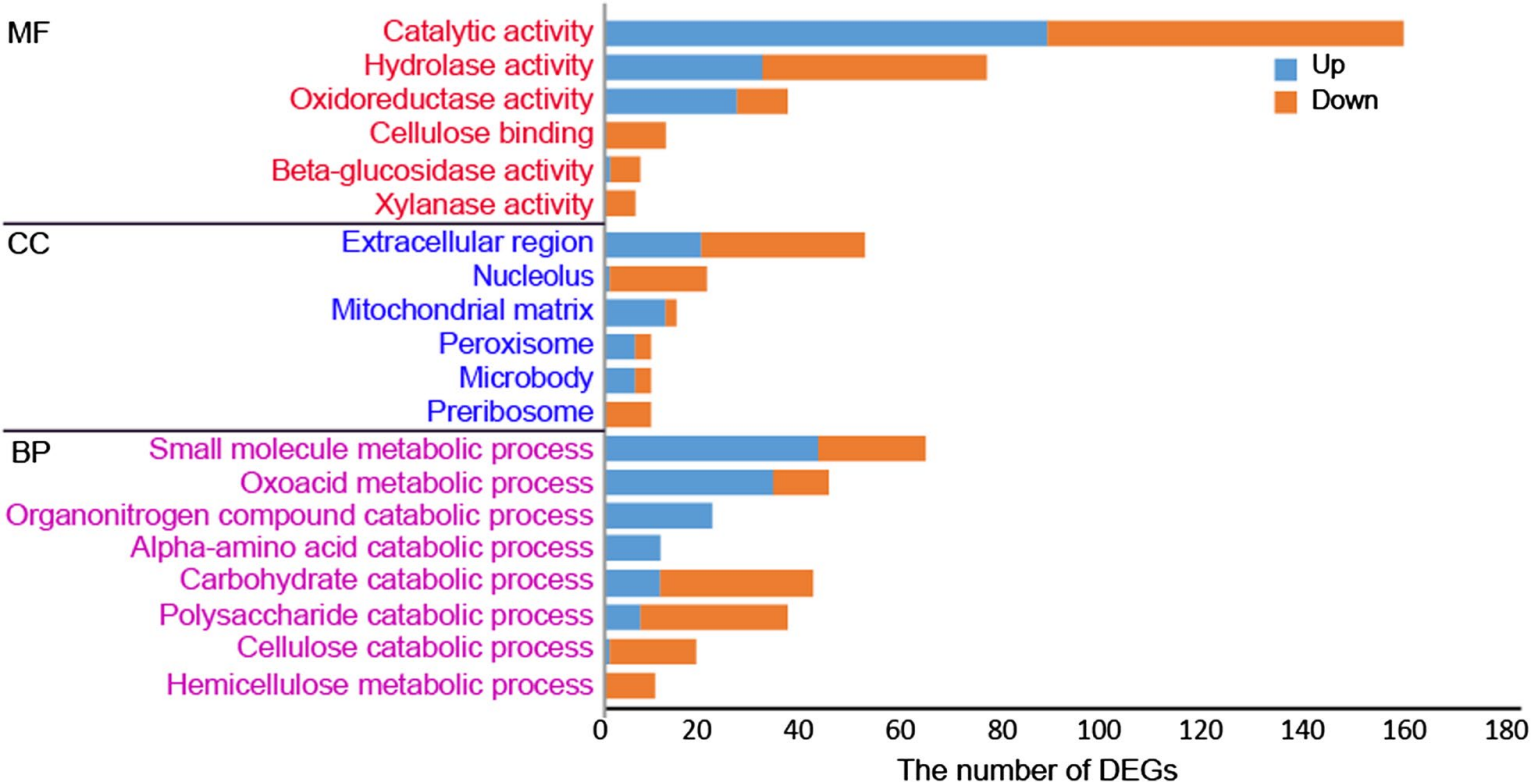
Gene ontology (GO) functional enrichment analysis of DEGs. The y axis represents the name of the most enriched GOs that belong to different ontologies. MF: the molecular function; CC: the cellular component; BP: the biological process

Gene ontology (GO) functional enrichment analysis of these DEGs showed that the most enriched molecular function was “catalytic activity”, which includes four enriched subcategories “hydrolase activity”, “oxidoreductase activity”, “β-glucosidase activity”, and “xylanase activity” (Fig. 4). The other enriched molecular function was “cellulose binding”. According to the analysis of the enriched cellular components, these DEGs were mainly distributed in extracellular region, nucleolus, mitochondrial matrix, peroxisome, microbody, and preribosome. For the enriched biological processes, the most enriched DEGs belong to “small molecule metabolic process” that includes two enriched subcategories “oxoacid metabolic process” and “alpha-amino acid catabolic process”, “organonitrogen catabolic process” and “carbohydrate catabolic process”, whose subcategory “polysaccharide catabolic process” was also enriched. The other two enriched biological processes were “cellulose catabolic process” and “hemicellulose catabolic process”, which belong to “polysaccharide catabolic process”. Most of DEGs in the enriched molecular functions “β-glucosidase activity”, “xylanase activity”, and “cellulose binding”, and the enriched biological processes “cellulose catabolic process” and “hemicellulose catabolic process” were downregulated, which were in line with the sharply decreased cellulase production after the addition of glutamine (Fig. 2A). Moreover, the enriched biological processes “organonitrogen catabolic process” and “alpha-amino acid catabolic process” indicate that the presence of glutamine affected the nitrogen metabolism of *T. reesei* grown on cellulose.

The top 29 enriched KEGG pathways were obtained by KEGG pathway enrichment analysis of DEGs (Additional file 9: Figure S1), which were classified into four categories: “metabolic pathway”, “biosynthesis of secondary metabolites”, “biosynthesis of antibiotics”, and “carbon metabolism”. The addition of glutamine majorly affected pathways in “amino acid metabolism” and “carbohydrate metabolism”. It seems that the metabolism of various amino acids including valine, leucine, isoleucine, tryptophan, cysteine, methionine, tyrosine and lysine was significantly changed by glutamine. (Hemi)cellulase-related genes belong to the enriched pathway “starch and sucrose metabolism”.

**Fig. 5:**
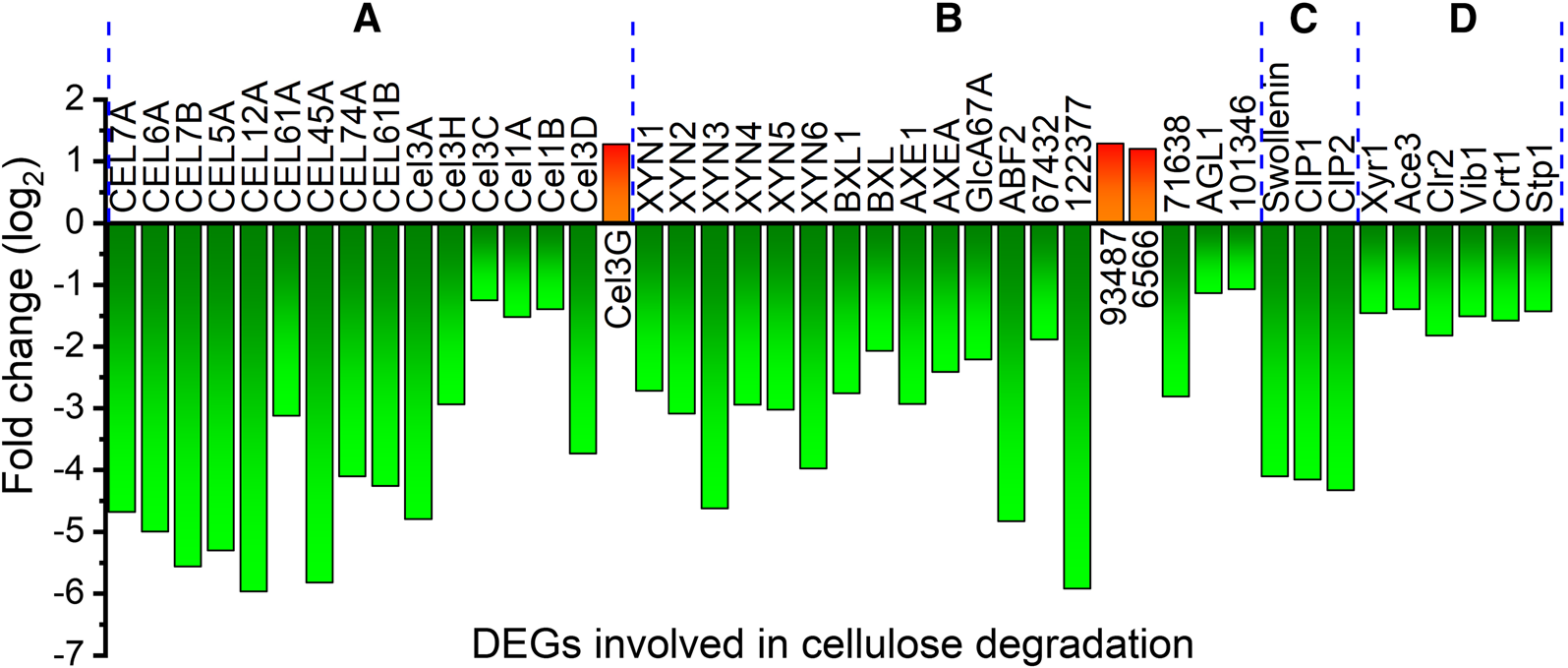
Relative transcription levels of DEGs involved in cellulose degradation including **A** cellulase, **B** hemicellulase, **C** nonenzymatic cellulose attacking enzymes, and **D** transcriptional factors

### Most DEGs involved in the cellulase production were decreased in glutamine-treated *T. reesei*

A total of 86 genes were known or predicted to be associated with (hemi)cellulase biosynthesis in *T. reesei*, of which 44 genes were DEGs with greatly reduced mRNA levels, except genes *cel3g*, M419DRAFT_93487 and M419DRAFT_6566 (Fig. 5 and Additional file 3: Table S3) that were upregulated*.* Particularly, the transcriptional levels of (hemi)cellulase genes including 2 cellobiohydrolases (CEL7A and CEL6A), 7 endoglucanases (CEL7B, CEL5A, CEL12A, CEL61A, CEL45A, CEL74A, and CEL61B), 6 β-glucosidases (CEL3A, CEL3H, CEL3C, CEL1A, CEL1B, and CEL3D), 6 xylanases (XYN1, XYN2, XYN3, XYN4, XYN5, and XYN6), and two β-xylosidases (BXL1 and BXL) were all reduced markedly, matching well with the decreased (hemi)cellulase activities as observed above. Genes encoding the auxiliary proteins like swollenin [26], and the cellulose-induced proteins Cip1 and Cip2 [27], which have been reported to enhance cellulose degradation, were also down-expressed markedly. Furthermore, 6 cellulase transcription factors were significantly downregulated, including the well-known cellulase transcription activators Xyr1 [28], Ace3 [29], Clr2 [30], and Vib1 [31, 32], and MFS sugar transporters Crt1 and Stp1 [33, 34]. We are surprised to find that in contrast to most genes related to cellulase synthesis, the mRNA levels of *cel3g*, M419DRAFT_93487 and M419DRAFT_6566 were increased with the addition of glutamine. However, the reason behind this phenomenon is unknown as these three genes are lack of study, which is worthy of research in future study. This suggests that cellulase productions are subject to nitrogen regulation at the transcriptional level.

### The impact of glutamine on nitrogen metabolism of *T. reesei*

15 DEGs are involved in ribosome biogenesis, all of which were downregulated except gene M419DRAFT_104236 (Additional file 4: Table S4). All these downregulated genes are related to the assembly and maturation of the large unit and small unit of ribosome. In contrast to the reduced mRNA levels of DEGs relevant to ribosomal biogenesis, all DEGs involved in amino acids were upregulated except that gene glutamate dehydrogenase (M419DRAFT_93755) was downregulated (Additional file 5: Table S5). Particularly, four of these DEGs are amino acid transporters, while the rest of genes are involved in branched or aromatic amino acid catabolic process.

**Fig. 6:**
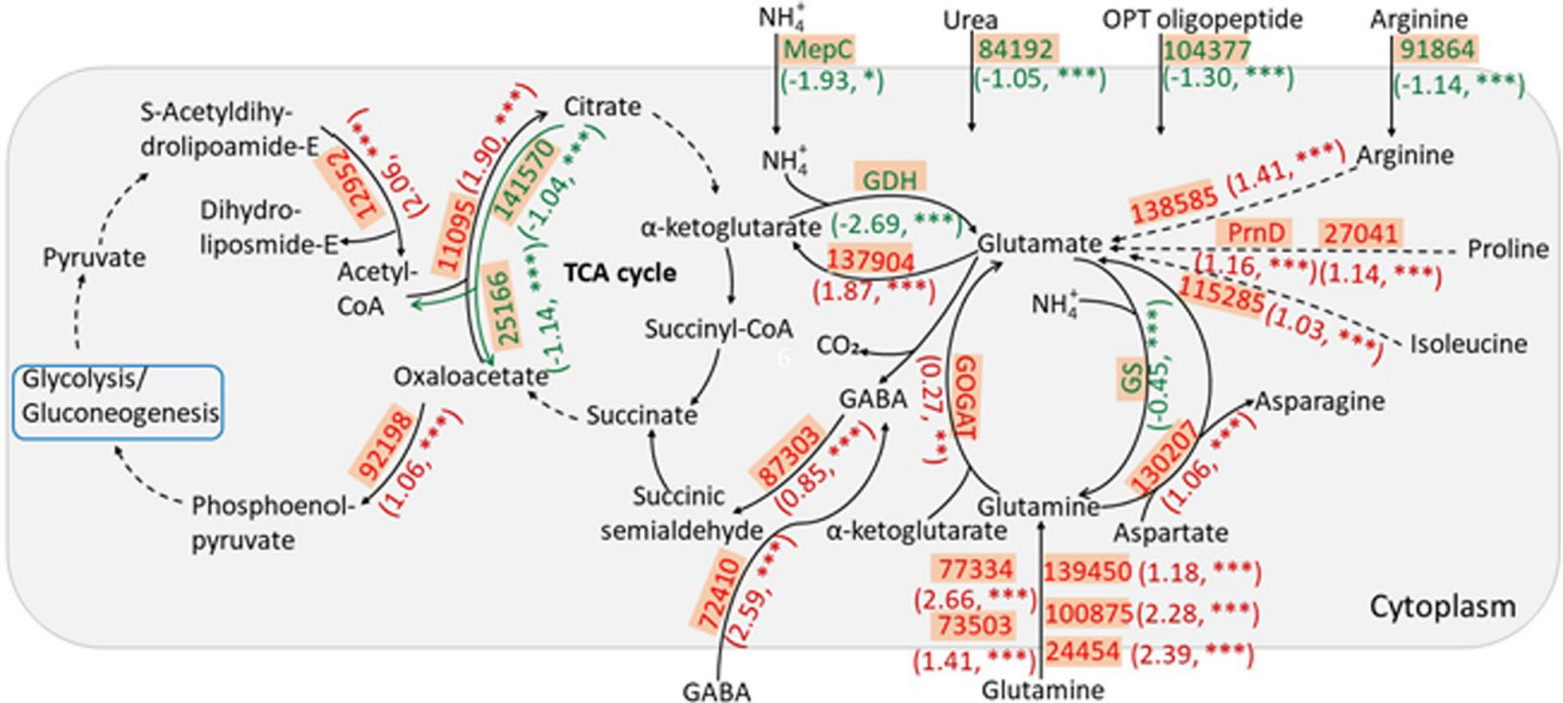
Effect of glutamine on the mRNA levels of genes involved in the nitrogen and carbon metabolism. The genes are highlighted with orange frames. The upregulated and downregulated genes are marked in red and green, respectively, with log_2_FC and *p* value in brackets. *p* < 0.05: *; *p* < 0.01: * *; *p* < 0.001: * * *. MepC (M419DRAFT_93825): ammonium permease; GDH (M419DRAFT_93755): glutamate dehydrogenase; GOGAT (M419DRAFT_24904): glutamate synthase; GS (M419DRAFT_101940): glutamine synthase; PrnD: proline oxidase; Other proteins were named based on the genome database of *T. reesei* (https://fungi.ensembl.org/Trichoderma_reesei_rut_c_30_gca_000513815/Info/Index)

In addition, a number of DEGs were found to be involved in nitrogen metabolism including the GS/GOAT cycle and the GABA shunt. Glutamate dehydrogenase (GDH) catalyzes the conversion of α-ketoglutarate (α-KG) and ammonia (NH_4_^+^) to glutamate. Then glutamine synthase (GS) uses glutamate and ammonia to synthesize glutamine. Both these genes were significantly downregulated as well as the ammonium permease (MepC) (Fig. 6), indicating glutamine biosynthesis was shut down in *T. reesei* treated with 2.92 g/L glutamine. In contrast, the glutamine catabolism was enhanced, as shown by the increased mRNA levels of GOGAT and M419DRAFT_130207. The addition of glutamine also led to an upregulation of amino acid permeases, such as M419DRAFT_73503, M419DRAFT_77334, M419DRAFT_139450, M419DRAFT_100875 and M419DRAFT_24454. Meanwhile, glutamate biosynthesis was enhanced as demonstrated by the significantly increased mRNA levels of GOGAT, M419DRAFT_138585, M419DRAFT_115285, and PrnD and M419DRAFT_27041, which were responsible for the conversion of glutamine, arginine, isoleucine and proline to glutamate, respectively. All these suggested that the addition of glutamine led to a markedly decreased glutamine biosynthesis and the significantly increased glutamate biosynthesis and glutamine catabolism. Moreover, the transporters for different nitrogen sources including ammonium (MepC), urea (M419DRAFT_84192), oligopeptide (M419DRAFT_84192), arginine (M419DRAFT_91864) were dramatically downregulated, while transporters for amino acids and 4-aminobutanoic acid (GABA), were significantly upregulated. The upregulated expression of amino acid transporters might be involved in the uptake of glutamine. Gene encoding GABA transaminase (M419DRAFT_87303) was also increased. GABA is an intermediate metabolite in the GABA shunt, a metabolic pathway that bypasses two enzymatic steps of the tricarboxylic acid (TCA) cycle to produce succinate from α-ketoglutarate via glutamate (Fig. 6).

As the GS/GOGAT cycle and the GABA shunt link primary nitrogen and carbon metabolism [1], the primary carbon metabolism might be also impacted by the addition of glutamine, as implied by the enriched glycolysis/ gluconeogenesis pathway (Additional file 9: Figure S1). Therefore, we also checked whether there were DEGs in TCA cycle and the glycolysis/gluconeogenesis pathway. 5 genes related to citrate cycle (TCA cycle, Krebs cycle) were differentially expressed. Acetyl-CoA was derived from pyruvate oxidation and then its acetyl group was transferred to oxaloacetate by citrate synthase to form citrate. The reaction rates of these two steps were speeded up as shown by the enhanced expression levels of M419DRAFT_12952 and M419DRAFT_11095. By contrast, the citrate lyase complex M419DRAFT_25166 and M419DRAFT_141570 were downregulated, which catalyzed the decomposition of citrate. Besides, the mRNA level of M419DRAFT_92198, which was responsible for the conversion of oxaloacetate to phosphoenolpyruvate was upregulated. As TCA cycle was a hub coordinating matter and energy conversion, the addition of glutamine possibly changed the pattern of mass transfer and energy utilization. Taken together, the presence of glutamine in TMM affected notably the nitrogen metabolism significantly by decreasing ribosomal biogenesis, glutamine biosynthesis and other nitrogen transport while increasing glutamate biosynthesis, amino acid catabolism, and glutamine catabolism. Glutamine also posed an effect on the carbon metabolism including TCA cycle and the glycolysis/ gluconeogenesis pathway.

### Genes *ooc1* and *ooc2* are associated with the cellulase repression impact of glutamine in *T. reesei*

**Fig. 7:**
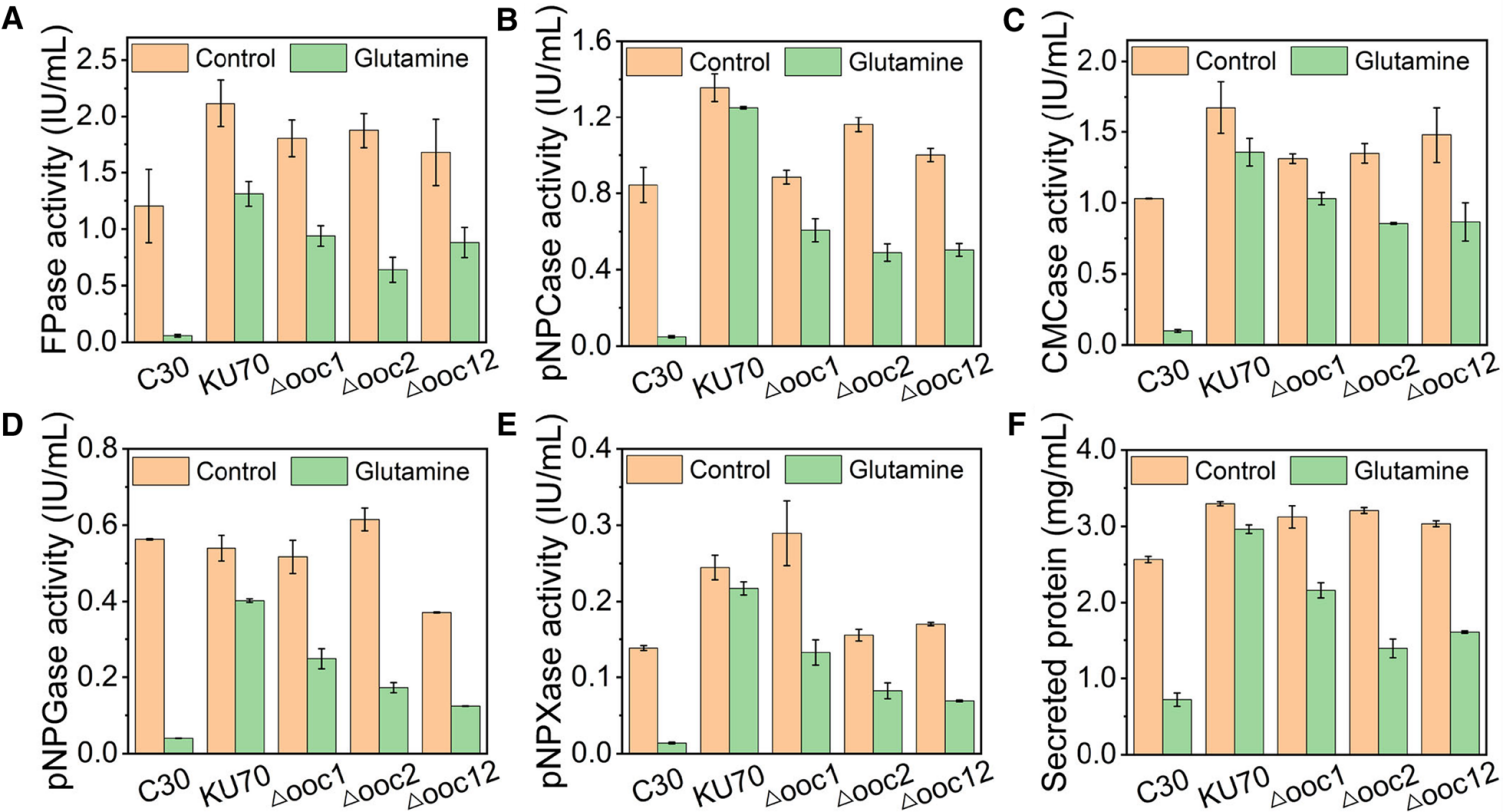
Impact of glutamine on (hemi)cellulase activities and protein secretion of *T. reesei* KU70, Δooc1, Δooc2, and Δooc12 cultured in TMM + 2% cellulose + 2.92 g/L glutamine for 120 h. **A** FPase: the filter paper activity; **B** pNPCase: the CBH activity; **C** CMCase: the CMC activity; **D** pNPGase: the β-glucosidase activity; **E** pNPXase: the β-xylosidase activity; **F** Secreted protein: secreted protein concentration. Data are represented as the mean of three independent experiments and error bars express the standard

It is worth noting that the transcriptional level of the reported cellulose signaling related protein ooc1 (M419DRAFT_124152) [35] was increased remarkably, and its isoenzyme (M419DRAFT_131885), which was named ooc2 here, was the most upregulated DEG in the presence of glutamine (Additional file 2: Table S2). Therefore, we are curious on the role of *ooc1* and *ooc2* in the responsiveness of *T. reesei* to glutamine. Genes *ooc1* and *ooc2* were deleted separately in *T. reesei* KU70, leading to recombinant strain Δooc1 and Δooc2, respectively. Strain KU70, a *ku70*-deleted derivative of RUT-C30, was utilized as the parent strain for its high efficiency of gene targeting [36]. Interestingly, we found that the inhibition effect of glutamine on cellulase production in strain KU70 was not that significant as that in RUT-C30, though a mild reduction of cellulase production after the treatment of glutamine was still observed (Fig. 7). It seems that the absence of gene *ku70* renders the resistance of *T. reesei* RUT-C30 to glutamine. Further deletion of *ooc1* and *ooc2* individually brought back the cellulase inhibition effect of glutamine in strain KU70 (Fig. 7), indicating *T. reesei* RUT-C30 increased the expression of genes *ooc1* and *ooc2* to antagonize the cellulase-depression effect of glutamine, but failed. In particular, the inhibition effect of glutamine on cellulase production in strain Δooc2 was worse than that in strain Δooc1, although the abundance of *ooc2* was far lower than that of *ooc1*, as shown by that the FPKM of *ooc2* was only 0.21% of that of *ooc1* in *T. reesei* under normal conditions (Additional file 2: Table S2). Moreover, in mutant strain Δooc12 with the double knockout of *ooc1* and *ooc2*, the inhibition effect of glutamine on cellulase production was increased as compared to that in strain Δooc1, but was similar to that in strain Δooc2, demonstrating gene *ooc1* was a functional redundancy to gene *ooc2*.

### The role of the TOR signaling pathway in glutamine-repressed cellulase production

**Fig. 8:**
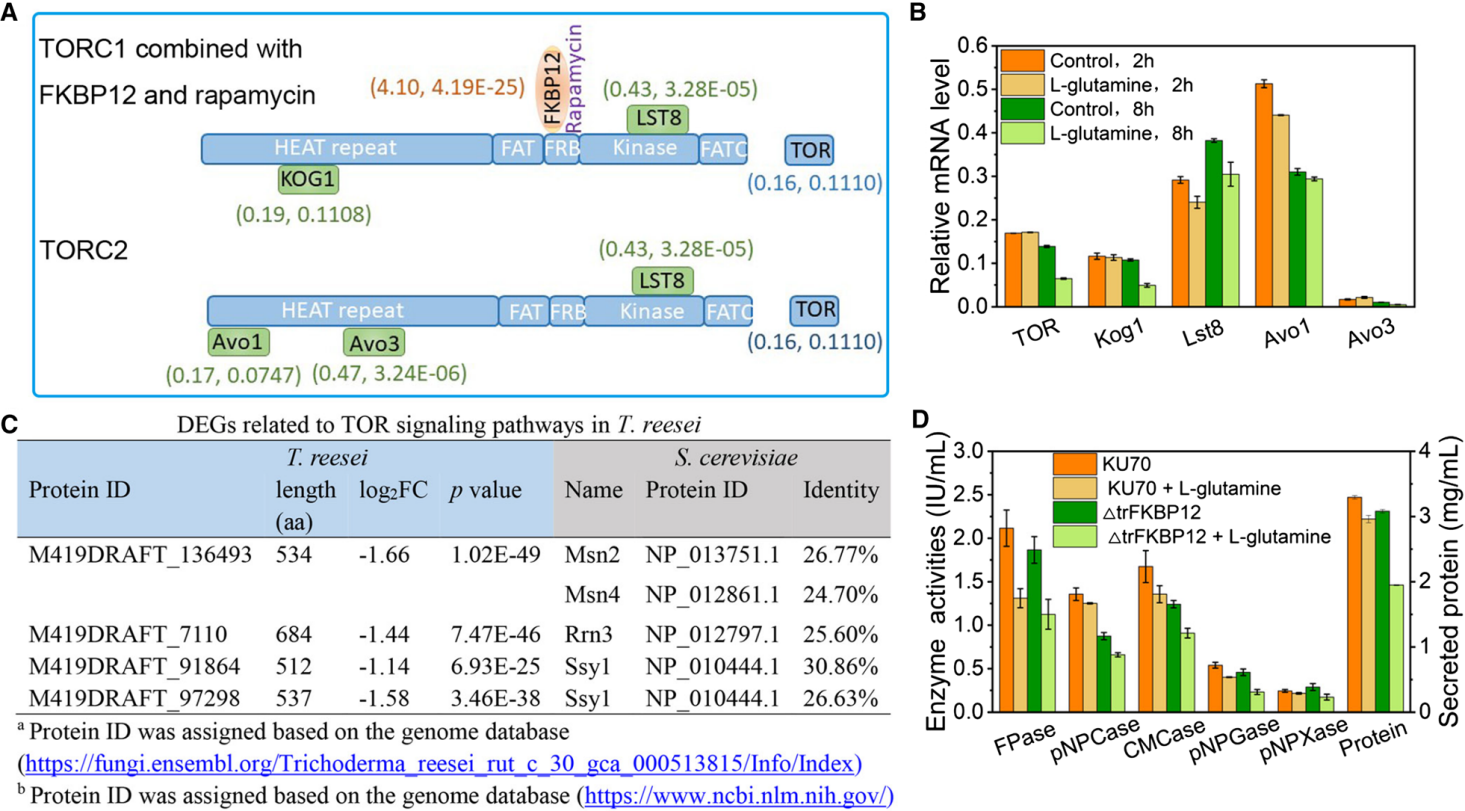
Impact of glutamine on the TOR signaling pathways. **A** Relative transcription levels of *trFKBP12*, and TORC1/2 components in the presence of 2.92 g/L glutamine for 24 h. Log_2_FC and *p* value were in brackets, respectively. **B** mRNA level of TORC1 and TORC2 components at 2 h and 8 h in *T. reesei* with or without 2.92 g/L glutamine. **C** DEGs related to TOR signaling pathways in *T. reesei* RUT-C30. **D** (Hemi)cellulase activities of strains KU70 and ΔtrFKBP12 at 120 h cultured in TMM + 2% cellulose with/without 2.92 g/L glutamine. Data are represented as the mean of three independent experiments and error bars express the standard

In mammals, yeasts and plants, the target-of-rapamycin (TOR) kinase signaling pathway was a central signaling hub integrating cell growth and metabolism [37–39]. It could be activated by amino acids, of which glutamine has been implicated in TOR activation [40]. Protein TOR contains conserved HEAT repeat, FAT, FKBP12/rapamycin binding (FRB), kinase, and FATC domains from the N to the C terminus, and forms two distinct physical and functional complexes, which are termed as TOR complex 1 (TORC1) and TORC2 (Fig. 8A). We wondered if the addition of glutamine into TMM could activate TOR and its related signaling pathways in *T. reesei*. Unexpectedly, transcriptional analysis showed that neither TOR nor the components of TORC1 and TORC2 were changed significantly after the treatment of glutamine for 24 h (Fig. 8A). Further analysis of their mRNA levels at 2 h and 8 h showed that TOR and Kog1 were significantly downregulated at 8 h, while other genes did not change obviously at either timepoints (Fig. 8B). For genes associated with TOR signal pathways including 15 genes for ribosome biogenesis, 8 genes for cell cycle/growth, 25 genes for nutrient uptake, 1 gene for stress, 5 genes for lipid metabolism, 6 genes for cell wall integrity, and 5 genes for autophagy [41] (Additional file 6: Table S6), only four genes (Rrn3, M419DRAFT_136493, M419DRAFT_91864 and M419DRAFT_97298) were differentially expressed (Fig. 8C). Rrn3 was a transcription initiation factor that regulated RNA polymerase (Pol) I-dependent gene expression during Pol I transcription initiation [42]. As Pol I was responsible for producing the 35S rRNA precursor, the downregulated Rrn3 might lead to reduced ribosome biogenesis, which was confirmed by the downregulated expression of genes involved in ribosome biogenesis as we observed above. The downregulated M419DRAFT_136493, designated as stress response element binding protein, was homologous to Msn2 and Msn4, which were zinc finger transcription factors that regulate the general stress response, such as oxidative stress, heat shock, osmotic stress, high ethanol concentration and nutrient depletion in *Saccharomyces cerevisiae* [43]. M419DRAFT_91864 and M419DRAFT_97298 homologous to Ssy1 functioning as the primary amino acid receptor [44] were also downregulated. All these demonstrated that TOR and the corresponding kinase signaling pathways were not very sensitive to glutamine in *T. reesei*. By contrast, the cellular receptor of rapamycin *trFKBP12* was noticeably upregulated by 2^4.10^ (17.14)-fold with 2.92 g/L glutamine. To see whether *trFKBP12* was involved in the negative impact of glutamine on cellulase production, the *trFKBP12* deletion strain ΔtrFKBP12 engineered in our previous study [41] was treated with glutamine. Similar to the deletion of genes *ooc1* and *ooc2*, the knockout of *trFKBP12* exacerbated the cellulase depression influence of glutamine (Fig. 8D), suggesting that the upregulated expression of *trFKBP12* by glutamine under nitrogen excess might also harnessed to antagonize the cellulase depression influence of glutamine. In summary, it seems that genes *ooc1*, *ooc2*, and *trFKBP12* are all involved in the cellulase depression influence of glutamine, which might fight against the negative effect by the upregulation of these genes.

## Discussion

To varying degrees, the supply of different nitrogen sources at equal amount to TMM culture medium inhibited cellulase production in *T. reesei* RUT-C30. Glutamate displayed the weakest inhibition effect on cellulase production, followed by ammonium sulfate, tryptone, urea, yeast extract and glutamine in ascending order. The cellulase inhibition was found regardless of nitrogen sources, while the inhibition degree was dependent on nitrogen types. This suggests that the cellulase inhibition was due to both the nitrogen excess induced by the addition of more nitrogen sources and the administered nitrogen source itself. Although the nitrogen content of different nitrogen sources varies greatly at 2.92 g/L, 40.0 mM for glutamine, 97.2 mM for urea, 44.2 mM for ammonium sulfate, and 19.8 mM for glutamate, there is no notable correlation between the nitrogen content and cellulase inhibition. For example, the inhibition effects of 2.92 g/L glutamine and ammonium sulfate on cellulase production differed greatly, although they contained a similar nitrogen content. Despite of the most severe inhibition for glutamine on cellulase production, the nitrogen content of glutamine was between those of glutamate (19.8 mM) and urea (97.2 mM). Clearly, the cellulase inhibition effect was related to the nitrogen type rather than the nitrogen content. Glutamine possessed the strongest cellulase repression effect among all the tested nitrogen sources. Although both ammonium sulfate and glutamine are preferred nitrogen sources, glutamine displayed much stronger cellulase repression than that of ammonium sulfate, indicating that ammonium sulfate is a more suitable nitrogen source for cellulase production in *T. reesei* than glutamine. Meanwhile, glutamine and glutamate displayed a huge difference in the inhibition degree on cellulase production, though they can be converted to each other through the GS/GOGAT cycle in cells. It seems that glutamine may well be the metabolite effector in nitrogen repression of cellulase synthesis, similar to the role of glucose plays in CCR.

The presence of more nitrogen sources into TMM might lead to excess nitrogen, repressing cellulase production. During nitrogen sufficiency, glutamine as a strong nitrogen source for *T. reesei* might be utilized in preference to other nitrogen sources. Therefore, the presence of glutamine caused a significant change on nitrogen transport and metabolism in *T. reesei.* We can see the supply of glutamine shut down the transport of other nitrogen sources including ammonium, urea, oligopeptide (M419DRAFT_84192), and arginine, but enhanced its transport through the upregulation of amino acid transporters (Fig. 6). Particularly, the assimilation of ammonium was reduced significantly. Meanwhile, the presence of glutamine in TMM affected notably the nitrogen metabolism by repressing ribosomal biogenesis and glutamine biosynthesis while enhancing glutamate biosynthesis, amino acid catabolism, and glutamine catabolism.

On the other hand, glutamine is commonly thought to be the pivotal metabolite for sensing cellular nitrogen sufficiency and activating nitrogen metabolite repression [45]. Filamentous ascomycetes possess three important nitrogen regulators, namely, the activator AreA, and two repressors AreB and NmrA [7, 46]. AreA activates a broad range of genes involved in the utilization of secondary nitrogen sources in the absence of glutamine and ammonium, while AreB regulates AreA activity to repress AreA-dependent nitrogen catabolic genes under carbon-limiting conditions. Another level of regulation of AreA activity involves its interaction with the co-repressor NmrA. In addition, NmrA appears to modulate AreA and AreB activities in response to the carbon status of the cell. All these genes were reduced significantly after the addition of glutamine (Additional file 7: Table S7), though they were not DEGs according to the stringent standard we used, indicating that nitrogen metabolite repression occurred after the treatment of glutamine.

In the presence of glutamine, *T. reesei* adjusts its metabolism for a high rate of glycolysis and directs carbon flux to respiration and fermentation for biosynthesis and energy production, while genes involved in utilization of alternative carbon sources are repressed in a Cre1-dependent manner as suggested by the upregulated expression of the carbon catabolite repressor Cre1 (Additional file 7: Table S7). Cre1 was shown to modulate genes encoding amino acid transporters *T. reesei* [47], and play a role in nitrogen catabolite repression in *A. nidulans* [48], suggesting that the role of Cre1 is not limited to CCR but has a cell-wide role to ensure fungal growth and survival in the presence of varied carbon and nitrogen sources. Furthermore, Vib1 was significantly reduced (Fig. 5), in agreement with that gene deletion of *vib1* in strain RUT-C30 decreased cellulase expression [31]. In *Neurospora crassa*, Vib1 does not directly modulate cellulase gene expression, but impacts the expression level of an essential cellulase regulator CLR2 [49] which was downregulated significantly in the presence of glutamine in our study (Fig. 5). Vib1 functions in repressing both glucose signaling and CCR under carbon-limited conditions, enabling a proper cellular response for plant biomass deconstruction and utilization. Vib1 is also required for extracellular protease secretion in response to both carbon and nitrogen starvation in *N. crassa* [50]. The effect of glutamine on the mRNA levels of these genes relevant to both nitrogen and carbon metabolism implicated that glutamine impacted not only the nitrogen metabolism, but also the carbon metabolism for cellulase production in *T. reesei*.

Filamentous fungi require sufficient nutrient availability to meet biological demand for cell growth. Glucose and glutamine are two primary carbon/nitrogen sources of the TCA cycle, macromolecule production, and ATP generation for proliferating cells [51]. The addition of glutamine broke the previous equilibrium of TCA cycle and led to an increased synthesis of α-ketoglutarate and succinic semialdehyde (Fig. 6). Besides, nitrogen catabolite repression (NCR) was activated in the presence of glutamine, which represses the expression of genes for the use of alternative and less preferred nitrogen sources like ammonium, urea, OPT oligopeptide and arginine (Fig. 6). It seems that NCR mediated by glutamine has a negative effect on cellulase production, similar to that caused by CCR in the presence of glucose. When cellulose was the sole carbon source, the decreased synthesis of cellulase at both transcription and protein levels led to a short supply of glucose, which contributed to the inhibition of the growth and sporulation of *T. reesei* (Fig. 3).

Genes *ooc1* and *ooc2* are next to each other and oriented tail to tail in *T. reesei* genome. They are small proteins with 101 and 104 amino acids separately and are homologous to each other with 43% sequence identity. Both of them contain signal peptides for secretion, suggesting that they could be secreted to the extracellular space. When *T. reesei* QM9414 was cultured at pH 6.0 induced by cellulose, the expression levels of *ooc1* and *ooc2* were also increased compared to that at pH 3.0 and pH 4.5, indicating that *ooc1* and *ooc2* were pH responsive [52]. It seemed that *ooc1* and *ooc2* are functionally equivalent. Compared to the parental strain QM9414, *ooc1* was upregulated in *T. reesei* Δ*phlp1* (phosducin-like protein) [53], Δ*pkac1* (protein kinase) [54] or Δ*cre1* [54] strains in darkness. In *Trichoderma harzianum* T34, *ooc1* was induced during its interactions with *Pythium ultimum* when *nox1* was overexpressed [55]. In this study, they were among the most upregulated DEGs in the presence of glutamine (Additional file 2: Table S2), whose knockout worsened the cellulase inhibition effect of glutamine in strain KU70 (Fig. 7). It seems that *T. reesei* RUT-C30 increased the expression of genes *ooc1* and *ooc2* to antagonize the cellulase-depression effect of glutamine. All these demonstrate that gene *ooc1* and *ooc2* in *T. reesei* RUT-C30 were highly responsive to various signals in the environment.

The TOR kinase, a central regulator of nutrient response, plays an important role in cell growth and metabolism and could be activated by glutamine [56]. However, TOR and TOR complexes were not differentially expressed with the addition of glutamine at 2 h, 8 h and 24 h, except that TOR and Kog1 were reduced at 8 h (Fig. 8B). By contrast, the mRNA level of *trFKBP12* was increased remarkably. This was similar to what we found previously that genes related to TOR signaling pathways were not changed significantly but *trFKBP12* was significantly upregulated by rapamycin in *T. reesei* grown on cellulose condition [41]. Nevertheless, there were still several genes in the TOR signaling pathways affected by glutamine, including the upregulated expression of the cellular receptor of rapamycin *trFKBP12,* and the downregulated expression of transcription initiation factor Rrn3, stress-responding transcription factors Msn2 and Msn4, as well as the primary amino acid receptor Ssy1. In addition, the deletion of *trFKBP12* reinforced the inhibition of glutamine on cellulase synthesis, showing that gene *trFKBP12* is related to the glutamine-inhibited cellulase production in *T. reesei*.

## Conclusion

Using *T. reesei* as an example, we first studied the effect of glutamine on cellulase-producing fungi under cellulose condition, including cellulase production, cell growth, sporulation, mycelium morphology and transcriptome. Under nitrogen excess condition, glutamine displayed the strongest inhibition effect on cellulase production, followed by ammonium sulfate, tryptone, urea, yeast extract and glutamine in descending order. Cellulase production was declined and the growth as well as sporulation were impaired with the addition of glutamine, while the mycelium morphology was not changed. Further RNA-seq analysis showed that genes associated with the cellulase production were decreased severely at the mRNA level in the presence of glutamine. Moreover, glutamine exerted a remarkable change on the transcriptional levels of genes related to nitrogen metabolism, such as the decreased expression of genes taking part in non-glutamine nitrogen transport, ribosomal biogenesis and glutamine biosynthesis, and the enhanced expression of genes associated with glutamate biosynthesis, amino acid catabolism, and glutamine catabolism. Genes *ooc1*, *ooc2* and *trFKBP12* are associated with the cellulase repression impact of glutamine in *T. reesei*. All together, we found that glutamine is probably the metabolite effector in nitrogen repression of cellulase synthesis by significantly changing nitrogen transport and metabolism, which is facilitated by genes *ooc1*, *ooc2*, and *trFKBP12*. These findings provide a comprehensive understanding of the regulatory mechanism of nitrogen in cellulase production in filamentous fungi, which would help in establishing optimal fermentation strategies for better cellulase production in industry.

## Methods

### Microbial strains, plasmids and cultivation conditions

*Escherichia coli* DH5α was used as the cloning host for plasmid construction. *Agrobacterium tumefaciens* AGL-1 was used as a T-DNA donor for fungal transformation. *T. reesei* RUT-C30 (CICC 13052, ATCC 56765) was purchased from China Center of Industrial Culture Collection. *T. reesei* KU70, where *ku70* was deleted in RUT-C30 [57], was provided friendly by Professor Wei Wang from East China University of Science and Technology. *E. coli* DH5α and *A. tumefaciens* AGL-1 were cultivated in Luria–Bertani (LB) with 220 rpm at 37 °C and 28 °C, respectively. *T. reesei* were grown on potato dextrose agar (PDA) plates for conidia production and in TMM [58] with 2% (w/t) cellulose for cellulase production at 28 °C with 220 rpm. The TMM medium was as followed: Tryptone, 0.75 g/L; Yeast extract, 0.25 g/L; Urea, 1.00 g/L; (NH_4_)_2_SO_4_, 4.00 g/L; KH_2_PO_4_, 6.59 g/L; Maleic acid, 11.6 g/L; FeSO_4_ * 7H_2_O, 0.005 g/L; MnSO_4_ * H_2_O, 0.0016 g/L; ZnSO_4_ * 7H_2_O, 0.0014 g/L; CoCl_2_ * 6H_2_O, 0.002 g/L; MgSO_4_, 0.60 g/L; CaCl_2_, 0.60 g/L; Tween 80, 0.186 ml/L [58]. When glutamine was the sole nitrogen source, different concentrations of glutamine were used to replace all the nitrogen sources of TMM. All chemicals used in this research were ordered from Sigma-Aldrich, USA.

### Shake flask cultivation

Five percent (v/v, 10^7^ /mL) conidia of *T. reesei* were inoculated into 10 mL sabouraud dextrose broth (SDB) and cultured at 28 °C with 200 rpm for 2 days. 5 mL pre-grown mycelia were inoculated into 50 mL TMM media (pH 6) plus 2% cellulose with different concentrations of glutamine and incubated at 28 °C with 200 rpm for 7 days. The stock solution of glutamine (100 g/L) was prepared in water. Samples were taken at indicated timepoints for (hemi)cellulase activity assay, confocal observation, biomass dry weight measurement, and RNA-seq analysis. The samples of *T. reesei* culture collected above were centrifuged at 8000 rpm for 30 min to remove the cell pellets, leading to the supernatants for (hemi)cellulase activities assay as described in our previous research [59–62]. If required, samples were centrifuged at 8000 rpm for 30 min to isolate the mycelia from the supernatant.

For *T. reesei* grown in TMM with the addition of various nitrogen sources as indicated in the text, 2.92 g/L nitrogen source was added into TMM. For *T. reesei* grown in culture medium using different nitrogen sources as the sole nitrogen source, all the nitrogen sources of TMM were removed and 4 g/L each nitrogen source was added.

### Measurement of the biomass dry weight of *T. reesei* grown on cellulose

The biomass of *T. reesei* grown in TMM + 2% cellulose with and without glutamine was indirectly determined by the amount of intracellular protein [63, 64]. In brief, harvested mycelia were suspended in 1 M NaOH and incubated for 2 h with frequent vortex. Then the protein concentration of the supernatant of the suspension was determined by the Modified BCA Protein Assay Kit (Sangon Biotech, Shanghai, China). The biomass dry weight was calculated assuming an average content of 0.32 g intracellular protein per g of dry cell mass.

### Analysis methods

(Hemi)cellulase activity assay, confocal imaging, spore counting, RNA-seq analysis, and RT-PCR were performed as described in our previous research [41, 65]. *T. reesei* RUT-C30 cultured for 24 h with or without 2.92 g/L glutamine were utilized for RNA-seq. Especially, confocal images of *T. reesei* were taken using a confocal microscope SP8 (Leica, Germany) with a 100 × oil immersion objective. The spores were counted by a hemocytometer under a confocal microscope SP8 with a 20 × oil immersion objective. The relative mRNA level was normalized using the housekeeping gene *sar1* [66]. All the primers used in the text are described in Additional file 8: Table S8.

### Deletion of genes *ooc1* and *ooc2* in *T. reesei* KU70

The upstream and downstream sequences (~ 1500 bp) of gene *ooc1*, *ooc2*, and *ooc1* and *ooc2* were separately amplified by PCR using genomic DNA of *T. reesei* KU70 as a template, and cloned into plasmid pXBthg at *Xho*I and at *Bam*HI using ClonExpress™ II One Step Cloning Kit (Vazyme, China), leading to plasmids pXBthg-ooc1, pXBthg-ooc2, and pXBthg-ooc12, respectively. Genes *ooc1* and *ooc2* can be deleted simultaneously as they are next to each other in *T. reesei* genome. The resulting plasmids pXBthg-ooc1, pXBthg-ooc2, and pXBthg-ooc12 were individually transformed into *T. reesei* KU70 separately by the *Agrobacterium tumefaciens*-mediated transformation (AMT) method using hygromycin B as a marker [67], yielding the deletion strains △ooc1, △ooc2, and △ooc12, respectively. The primers used were listed in Additional file 8: Table S8 and the verification of gene deletion in the recombinant strains was confirmed by PCR (Additional file 9: Figure S2) and sequencing.

## Supplementary Information

**Additional file 1: Table S1.** The comparative transcriptome in *T. reesei* RUT-C30 cultured in TMM with/without the addition of glutamine.

**Additional file 2: Table S2.** Total DEGs in *T. reesei* RUT-C30 cultured in TMM with the addition of glutamine.

**Additional file 3: Table S3.** Genes involved in (hemi)cellulase production in *T. reesei* RUT-C30.

**Additional file 4: Table S4.** DEGs related to “ribosome biogenesis” in *T. reesei* RUT-C30 cultured in TMM with the addition of glutamine.

**Additional file 5: Table S5.** DEGs involved in amino acids in *T. reesei* RUT-C30 cultured in TMM with the addition of glutamine.

**Additional file 6: Table S6.** Genes involved in TOR signal pathways in *T. reesei* RUT-C30.

**Additional file 7: Table S7.** Genes involved in nitrogen and carbon catabolite repression in *T. reesei* RUT-C30.

**Additional file 8: Table S8.** Primers for gene cloning, PCR confirmation and qPCR.

**Additional file 9: Figure S1.** Kyoto Encyclopedia of Genes and Genomes (KEGG) enrichment analysis of DEGs. The y axis represents the name of the most enriched pathways. **Figure S2.** PCR confirmation of recombinant *T. reesei* strains △ooc1, △ooc2, and △ooc12.

## Data Availability

The data sets supporting the conclusions of this article are included in the article and its Additional files.

## Abbreviations

CCR: Carbon catabolite repression
NCR: Nitrogen catabolite repression
TMM: *Trichoderma* Minimal medium
GDH: Glutamate dehydrogenase
α-KG: α-ketoglutarate
NH_4_^+^: Ammonia
GS: Glutamine synthase
MepC: Ammonium permease
pNPGase: The β-glucosidase activity
pNPCase: The CBH activity
CMCase: The CMC activity
FPase: The filter paper activity
pNPXase: The β-xylosidase activity
AMT: *Agrobacterium*-mediated fungal transformation
SDB: Sabouraud dextrose broth
PDA: Potato dextrose agar
