# Supplementary material for: Glutamine involvement in nitrogen regulation of cellulase production in fungi
Source: Biotechnol Biofuels. 2021 Oct 13;14:199. doi: 10.1186/s13068-021-02046-1 (PMC8513308; doi:10.1186/s13068-021-02046-1)
Supplement: Supplementary file 8 — Additional file 8: Table S8. Primers for gene cloning, PCR confirmation and qPCR. [file 13068_2021_2046_MOESM8_ESM.docx]

Additional file 8：

Table S8 Primers for gene cloning, PCR confirmation and qPCR

| Primer | Sequence (5’– 3’) |
| --- | --- |
| **For deletion of genes *ooc1*, *ooc2*, and *ooc12*** | |
| Ooc1-UP-F | ATTATTATGGAGAAACTCGAGTGCCTGGTGTTGAAGTCGTGTA |
| Ooc1-UP-R | CCGTCACCAGCCCTGCTCGAGTTTTGTTCGTAGTAGGATAAAAAAGTC |
| Ooc1-DO-F | GTGAGGGTTAATTGCGCGGATCCAGGTGGAGTGGTTTTATTGCG |
| Ooc1-DO-R | CAGGTCGACTCTAGAGAGGATCCTATAAGCCCAGGCCTTTACCT |
| Ooc-UP-F | ATTATTATGGAGAAACTCGAGGGCCGATATGTAAGATGTGAAGAAGT |
| Ooc2-UP-R | CCGTCACCAGCCCTGCTCGAGTCTGATCTCTTGTTGAAATGACCAAG |
| Ooc2-DO-F | GTGAGGGTTAATTGCGCGGATCCAGCTATCGAACCTTTTCTAGTTCTAG |
| Ooc2-DO-R | CAGGTCGACTCTAGAGAGGATCCGCGTATTGAGACACTTGCCAAG |
| Ooc12-UP-F | ATTATTATGGAGAAACTCGAGTGCCTGGTGTTGAAGTCGTGTA |
| Ooc12-UP-R | CGTCACCAGCCCTGCTCGAGTTTTGTTCGTAGTAGGATAAAAAAGTC |
| Ooc12-DO-F | GTGAGGGTTAATTGCGCGGATCCTCTGATCTCTTGTTGAAATGACCAAG |
| Ooc12-DO-R | CAGGTCGACTCTAGAGGATCCGGCCGATATGTAAGATGTGAAGAAGT |
|  | |
| **For PCR confirmation of *T. reesei* mutants Δooc1, Δooc2, and Δooc12** | |
| Ooc1-F | ATGTTCTCCTATCTTGCCGG |
| Ooc1-R | GTGCACATATCGTTGACGGT |
| Ooc2-F | TTACATGACGCACTGGTCGT |
| Ooc2-R | AGTACCTGGGTTCCTCTCTTTG |
| Ooc12-F | TTACATGACGCACTGGTCGT |
| Ooc12-R | AGTACCTGGGTTCCTCTCTTTG |
|  |  |
| **For qPCR** | |
| FKBP12-F | CTCGGAGCGACACCATCAAG |
| FKBP12-R | CGTCCTTCGGTCGTATGAGG |
| TOR-F | CTTGCGCCAAGAGACCATCA |
| TOR-R | CGGGGTCAATCCAGTCATCA |
| Kog1-F | GCGCCAGACATGGCTTTGA |
| Kog1-R | GGCGCCAATCTTGGAGTTC |
| Lst8-F | AGCTCATTCCCGAGGAGGAC |
| Lst8-R | GAGCGCTGAACTGCGTTACC |
| Avo1-F | GGGTGTAAGGTGCCTCGAAA |
| Avo1-R | TTCAGCTCCGCCACAATCTC |
| Avo3-F | GGCTATCGTGCGACGAGATA |
| Avo3-R | CTTGAGAGCCTGCTCTCGTT |
