# Supplementary material for: Glutamine involvement in nitrogen regulation of cellulase production in fungi
Source: Biotechnol Biofuels. 2021 Oct 13;14:199. doi: 10.1186/s13068-021-02046-1 (PMC8513308; doi:10.1186/s13068-021-02046-1)
Supplement: Supplementary file 9 — Additional file 9: Figure S1. Kyoto Encyclopedia of Genes and Genomes (KEGG) enrichment analysis of DEGs. The y axis represents the name of the most enriched pathways. Figure S2. PCR confirmation of recombinant T. reesei strains △ooc1, △ooc2, and △ooc12. [file 13068_2021_2046_MOESM9_ESM.docx]

**Glutamine Involvement in Nitrogen Regulation of Cellulase Production in Fungi**

**Ai-Ping Pang,^1^ Funing Zhang,^1^ Xin Hu,^1^ Yongsheng Luo,^1^ Haiyan Wang,^1^ Samran Durrani,^1^ Fu-Gen Wu,^1^ Bingzhi Li,^2^ Zhihua Zhou,^3^ Zuhong Lu,^1*^ Fengming Lin,^1*^**

^1^State Key Laboratory of Bioelectronics, School of Biological Science and Medical Engineering, Southeast University, Nanjing, China

^2^Key Laboratory of Systems Bioengineering (Ministry of Education), School of Chemical Engineering and Technology, Tianjin University, Tianjin, China

^3^Key Laboratory of Synthetic Biology, Institute of Plant Physiology and Ecology, Shanghai Institutes for Biological Sciences, Chinese Academy of Sciences, Shanghai, China

*Correspondence: linfengming@seu.edu.cn; zhlu@seu.edu.cn


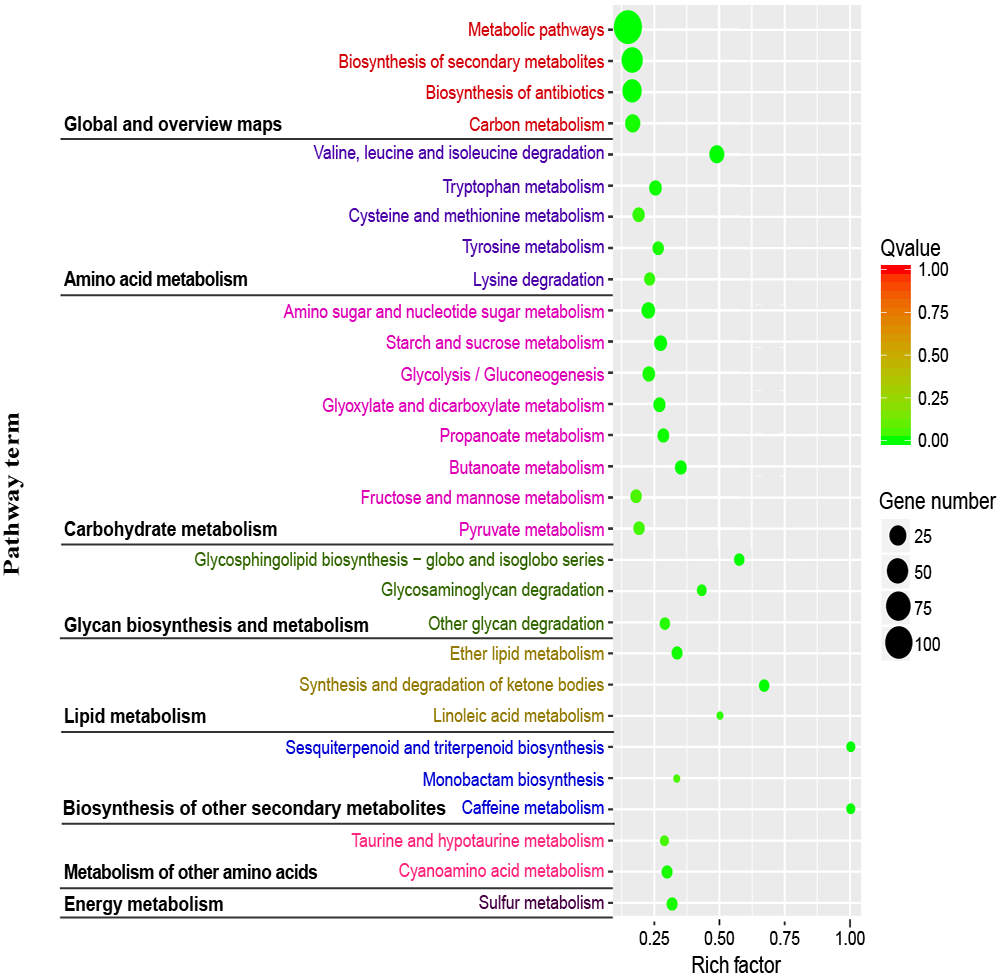


Figure S1 Kyoto Encyclopedia of Genes and Genomes (KEGG) enrichment analysis of DEGs. The y axis represents the name of the most enriched pathways.


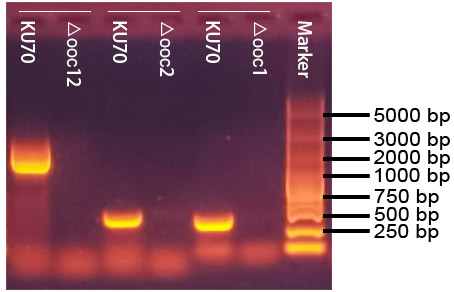


Figure S2 PCR confirmation of recombinant *T. reesei* strains △ooc1, △ooc2, and △ooc12. *T. reesei* KU70 was used as the negative control.
